# A Quantitative Prioritisation of Human and Domestic Animal Pathogens in Europe

**DOI:** 10.1371/journal.pone.0103529

**Published:** 2014-08-19

**Authors:** K. Marie McIntyre, Christian Setzkorn, Philip J. Hepworth, Serge Morand, Andrew P. Morse, Matthew Baylis

**Affiliations:** 1 Department of Epidemiology and Population Health, Institute of Infection and Global Health, University of Liverpool, Leahurst Campus, Neston, Cheshire, United Kingdom; 2 National Consortium for Zoonosis Research, Leahurst, Neston, Cheshire, United Kingdom; 3 Institut des Sciences de l'Evolution, Centre National de la Recherche Scientifique (CNRS), Université Montpellier 2, Montpellier, France; 4 Unité de recherche (UR) Animal et Gestion Intégrée des Risques (AGIRs), La Recherche Agronomique pour le Développement/Agricultural Research for Development (CIRAD), Montpellier, France; 5 School of Environmental Sciences, Roxby Building, University of Liverpool, Liverpool, United Kingdom; 6 NIHR Health Protection Research Unit in Emerging Infections (including Zoonoses) and Biological Threats, University of Liverpool, Liverpool, United Kingdom; Kliniken der Stadt Köln gGmbH, Germany

## Abstract

Disease or pathogen risk prioritisations aid understanding of infectious agent impact within surveillance or mitigation and biosecurity work, but take significant development. Previous work has shown the H-(Hirsch-)index as an alternative proxy. We present a weighted risk analysis describing infectious pathogen impact for human health (human pathogens) and well-being (domestic animal pathogens) using an objective, evidence-based, repeatable approach; the H-index. This study established the highest H-index European pathogens. Commonalities amongst pathogens not included in previous surveillance or risk analyses were examined. Differences between host types (humans/animals/zoonotic) in pathogen H-indices were explored as a One Health impact indicator. Finally, the acceptability of the H-index proxy for animal pathogen impact was examined by comparison with other measures. 57 pathogens appeared solely in the top 100 highest H-indices (1) human or (2) animal pathogens list, and 43 occurred in both. Of human pathogens, 66 were zoonotic and 67 were emerging, compared to 67 and 57 for animals. There were statistically significant differences between H-indices for host types (humans, animal, zoonotic), and there was limited evidence that H-indices are a reasonable proxy for animal pathogen impact. This work addresses measures outlined by the European Commission to strengthen climate change resilience and biosecurity for infectious diseases. The results include a quantitative evaluation of infectious pathogen impact, and suggest greater impacts of human-only compared to zoonotic pathogens or scientific under-representation of zoonoses. The outputs separate high and low impact pathogens, and should be combined with other risk assessment methods relying on expert opinion or qualitative data for priority setting, or could be used to prioritise diseases for which formal risk assessments are not possible because of data gaps.

## Introduction

Disease or pathogen risk prioritisation exercises are used by organisations charged with providing surveillance and mitigation measures including disease management and control, and biosecurity measures. Qualitative, semi-quantitative or quantitative approaches can be used, but most take significant time to develop, so their use is limited, and when research involving the study of multiple diseases or pathogens is planned, agents are rarely systematically selected.

Quantitative measures for risk prioritisation include the calculation of epidemiological parameters such as disease incidence, prevalence, mortality and morbidity rates, costs of prevention, treatment or control, and for human disease, years lived with disability (YLD) and disability-adjusted-life-year estimates (DALY). Additional measures for animals include losses to production. For many diseases, robust estimates of these measures do not exist. Semi-quantitative and qualitative risk assessments are less demanding of data than quantitative approaches. Nevertheless, they require significant time and physical resources (for example, to obtain parameters and effect sizes from the scientific literature), need updating regularly, and they usually require expert-opinion, adding subjectivity [1], [2], [3], [4], [5].

The H-index is an alternative approach to disease prioritisation. It objectively and rapidly provides a quantitative proxy of human disease or pathogen impact [6],(McIntyre, *unpublished*). The H-index captures scientific interest in a disease by deriving a metric from the number of papers published and how many citations each receives. Combining scientific impact (citations) with technical productivity (papers published) is useful as, individually, total papers does not account for the quality of publications, while citation count may be influenced by a small number of seminal papers or if a disease becomes ‘fashionable’ briefly. The H-index method is significantly correlated with more comprehensive measures of human infectious disease impact, including DALYs [6], and deaths from disease (McIntyre, *unpublished data*). It can be rapidly obtained at low cost, attained automatically, and repeated regularly to reflect changes in impact, serving as a generic tool to assess the relative bearing of diseases or pathogens, in an easier, timelier manner than traditional risk assessments. While the H-index method undoubtedly has limitations, these tend to be different to those of other approaches; its use could be a step forward in separating high and low priority diseases or pathogens, in combination with other risk assessment methods.

The ENHanCEd Infectious Diseases (EID2) database integrates published data sources on pathogens, their hosts (including vectors) and geographic ranges [7]. By coupling the H-index method with the EID2, the primary aim of this study was to establish priority lists of human and domestic animal pathogens (including zoonoses) present in Europe. We then consider reasons for the omission of some pathogens in our lists from those of other disease prioritisations: the 2010 Global Burden of Disease (GBD) estimates [8], communicable human diseases reportable in the European Community [9], the OIE list of notifiable animal diseases, infections and infestations [10], and the EU FP7 DISCONTOOLS project [11]. The GBD 2010 study was a large collaborative five year project which used all relevant published and unpublished evidence to create the strongest evidence-based epidemiological assessment of people's infectious and non-infectious health problems around the world [8]. The DISCONTOOLS project, funded by the European Commission over five years, investigated the impacts of 52 domestic animal diseases, to focus and prioritise future research [11]. As the zoonotic and emerging status of pathogens as well as their taxonomic division could affect the likelihood of their inclusion in surveillance and impact quantification work, these factors were also investigated as reasons for omission from the other disease prioritisations.

The H-index can be obtained in the same way for both human and animal pathogens. It therefore has potential as a single metric for prioritising across both host groups. Its potential as a quantitative One Health indicator (i.e. a single measure applicable to both human and animal diseases) was investigated by comparing scores for human-only, zoonotic, and animal-only pathogen groups, including emerging status as this would likely drive research impact.

Previous work has shown that the H-index is a proxy for human disease impact [6],(McIntyre, *unpublished*). We investigated its value as a proxy for animal disease impact by comparing domestic animal pathogen H-indices with other measures of impact including presence on the OIE list [10], and inclusion in DISCONTOOLS [11].

## Methods

### EID2 pathogen information

The EID2 database collates data on human and domestic animal pathogens: where, when, and in which hosts there is evidence of their occurrence. The database is built largely using automated procedures to interrogate publicly available databases. An EID2 background has been described previously [7]; here, we used similar criteria to define pathogens, including pathogenic status (frequently pathogenic: a pathogen which frequently causes a clinically pathogenic effect - morbidity or mortality - in humans or domestic animals; non-pathogenic: an organism which causes no clinical signs within any of its hosts; unknown pathogenicity: an organism for which there is insufficient evidence to decide), evidence of pathogens affecting hosts (‘host-pathogen interactions’: evidence from at least one piece of meta-data uploaded with DNA or RNA sequence information to [12], which describes where, when and from which host the pathogen came, or specific scientific publications [13]), and evidence of pathogens occurring within countries (evidence from at least one piece of meta-data [12], or at least five publications in [13] where pathogen name and a country MeSH-term [14] co-occurred in the title/abstract). Information on host-pathogen interactions was collated when there was evidence of a pathogen occurring in at least one host of interest to the study (including humans and European domestic animals; see Table 1). Further information about each organism, such as their taxonomic division for pathogens (bacteria – including rickettsia, fungi – including algal pathogens, helminths – including thorny-headed worms and pentastomids, protozoa, and viruses – including prion agents) or their taxonomic rank (genus, species, etc.) is stored using a series of statements. Previously, we examined characteristics of pathogen species [7]; here, we include sub-species, to account for important strains e.g. *Escherichia coli* O157∶H7.

**Table 1:** Animal species including humans for which pathogens have been studied, including domestic animals we eat or companion animals we keep as pets, and exotic animals also used as food sources or as pets.

Scientific name	Common name	Scientific name	Common name
*Agapornis personata*	Masked lovebird	*Lama glama*	Lama
*Agapornis roseicollis*	Rosy-faced lovebird	*Lama pacos*	Alpaca
*Anas platyrhynchos*	Domestic duck	*Meleagris gallopavo*	Turkey
*Anser anser*	Domestic goose	*Melopsittacus undulatus*	Budgerigar
*Bison bison*	American bison	*Meriones unguiculatus*	Mongolian gerbil
*Bison bonasus*	European bison	*Mesocricetus auratus*	Syrian golden hamster
*Bos indicus*	Zebu	*Mus musculus*	House mouse
*Bos taurus*	Cow	*Mustela putorius furo*	Domestic ferret
*Camelus dromedarius*	Dromedary	*Numida meleagris*	Helmeted guineafowl
*Canis lupus familiaris*	Domestic dog	*Nymphicus hollandicus*	Cockatiel
*Capra hircus*	Domestic goat	*Oryctolagus cuniculus*	Domestic rabbit
*Capreolus capreolus*	Roe deer	*Ovis aries*	Sheep
*Cavia porcellus*	Domestic guinea pig	*Ovis aries musimon*	Mouflon
*Cervus elaphus*	Red deer	*Pavo cristatus*	Blue peafowl
*Chinchilla lanigera*	Chinchilla	*Phasianus colchicus*	Ring-necked pheasant
*Columba livia*	Domestic pigeon	*Rangifer tarandus*	Reindeer
*Cricetus cricetus*	Common hamster	*Rattus norvegicus*	Brown rat
*Dama dama*	Fallow deer	*Rattus rattus*	Black rat
*Equus asinus*	Domestic donkey	*Rhombomys opimus*	Great Gerbil
*Equus caballus*	Domestic horse	*Serinus canaria*	Canary
*Felis catus*	Domestic cat	*Struthio camelus*	Ostrich
*Gallus gallus*	Chicken	*Sus scrofa*	Wild boar
*Homo sapiens*	Humans	*Sus scrofa domesticus*	Domestic pig
*Lagopus lagopus scotica*	Red grouse		

### Emerging/zoonotic pathogen status

Information on whether pathogens were zoonotic, non-zoonotic, emerging and not emerging was examined based upon previously published information [15], [16]. If not included in earlier work or if their status had changed due to more recent scientific evidence, updated pathogen information was based upon the previous definitions. Zoonotic pathogens were classified as those naturally transmitted between vertebrate (non-human) animals and humans (as the definitive host), not including species which have recently evolved from animal pathogens but are no longer transmitted between animals and humans [15], [17]. Emerging pathogens are those that have appeared in a host population for the first time (including newly-evolved strains), or have occurred previously but are increasing in incidence or expanding into areas where they had not previously been reported [15], [17]. Pathogens needed to have emerged in several geographically distinct areas to be ‘emerging’.

### H-index literature search protocol

#### Information sources

H-index searches were undertaken in January 2012 using Web of Science (WoS) [18]. Previous work established that results of H-index searches for pathogens undertaken using different bibliographic sources (e.g. WoS, SCOPUS, Google Scholar) are not identical but are highly correlated [6].

#### Eligibility criteria

Searches were restricted to the years 1900 to 2010, inclusive. English is used in WoS, however searches also include foreign-language publication title translations. All literature in the WoS database has been published.

#### Searches

Searches were undertaken using search phrases specified in quotation marks (“”), the ‘topic’ search field and with no lemmatization. Phrases were compiled including pathogen scientific name, alternative names, synonyms and alternative spellings according to NCBI Taxonomy [19]. H-indices for clinical diseases used clinical terms as well as pathogen phrases for the main pathogens of disease. Virus searches also included synonyms and acronyms from the NCBI Taxonomy database and International Committee on Taxonomy of Viruses [19], [20], and the term ‘virus’, and excluded other entities (viral or non-viral) which shared acronyms. The Boolean operators ‘AND’, ‘OR’, and ‘NOT’ linked multiple search phrases.

#### Example of a search phrase

(“mycobacterium tuberculosis” OR “bacillus tuberculosis” OR “bacterium tuberculosis” OR “mycobacterium tuberculosis typus humanus” OR “mycobacterium tuberculosis var. hominis”).

### Pathogen prioritisation framework

**Figure 1:**
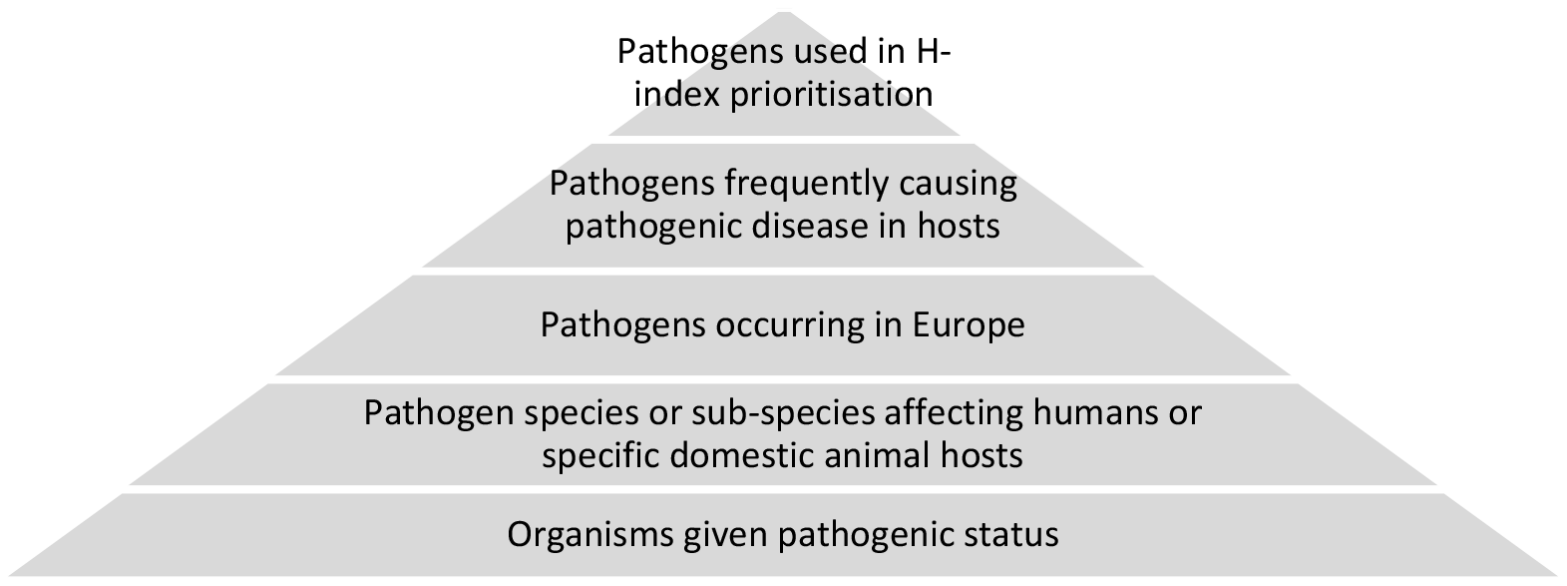
Pyramid diagram showing the prioritisation framework for pathogens leading to the use of the quantitative H-index methodology to estimate relative pathogen impact.

A full list of human and domestic animal pathogens frequently causing pathogenic effects and for which there was evidence of European occurrence was created using EID2 information [7], and defined criteria, see Figure 1. The relative impact of pathogens in this full list was assessed by calculating H-indices using the specified search protocol; high impact pathogens had the highest H-indices. The list was split according to host-pathogen interaction information, into two directories, one including pathogens with evidence of their occurrence in humans, and the second including domestic animal occurrence; zoonotic pathogens appeared in both lists. Information was manually obtained on whether these pathogens cause diseases featuring in other prioritisation lists [8], [10], [11], by examining pathogens listed under each disease's details in the NCBI MeSH library [14]; specific lists of diseases had been provided in other work [9], [11]. These additional pieces of information are included in the results (Tables 2 and 3). Finally, information on the pathogenic status of each pathogen, whether they frequently occurred in the relevant hosts and in Europe was verified by the study authors using manual literature searches of the scientific literature, for the pathogens with the highest H-indices.

**Table 2:** Top 100 human pathogens in Europe, prioritised according to the H-index methodology [6].

Pathogen name	H-index score	Taxonomic division	Pathogen name	H-index score	Taxonomic division
*Escherichia coli*, _Z, E, A, GBD_	524	Bacteria	*Streptococcus pyogenes*, _Z, E_	113	Bacteria
Human Immunodeficiency Virus 1, _NZ, E, GBD, EC_	410	Viruses	*Bacillus cereus*, _Z, NE, A_	111	Bacteria
Human Immunodeficiency Virus 2, _NZ, E, GBD, EC_	399	Viruses	*Aspergillus niger*, _Z, NE_	110	Fungi
Hepatitis C Virus, _NZ, E, GBD, EC_	289	Viruses	*Burkholderia cepacia*, _NZ, NE_	107	Bacteria
*Staphylococcus aureus*, _Z, E, A_	271	Bacteria	*Clostridium botulinum*, _Z, E, A, EC_	106	Bacteria
Human Herpesvirus 4, _NZ, NE_	257	Viruses	Encephalomyocarditis Virus, _Z, NE, A_	105	Viruses
*Helicobacter pylori*, _Z, NE, A_	246	Bacteria	*Yersinia pestis*, _Z, E, A, EC_	105	Bacteria
Hepatitis B Virus, _NZ, E, GBD, EC_	246	Viruses	*Streptococcus mutans*, _NZ, NE_	104	Bacteria
*Pseudomonas aeruginosa*, _Z, E, A_	243	Bacteria	*Aggregatibacter actinomycetemcomitans*, _Z, NE_	101	Bacteria
*Mycobacterium tuberculosis*, _Z, E, GBD, EC_	238	Bacteria	*Clostridium perfringens*, _Z, NE, A_	101	Bacteria
Human Papillomavirus, _NZ, E_	235	Viruses	*Serratia marcescens*, _Z, E_	100	Bacteria
*Bacillus subtilis*, _Z, NE_	219	Bacteria	*Yersinia pseudotuberculosis*, _Z, NE, A, EC_	99	Bacteria
*Listeria monocytogenes*, _Z, E, A, EC_	207	Bacteria	*Entamoeba histolytica*, _Z, NE, A_	98	Protozoa
*Streptococcus pneumoniae*, _Z, E, GBD, EC_	199	Bacteria	*Leishmania donovani*, _Z, E, A, GBD_	98	Protozoa
*Candida albicans*, _Z, E, A_	181	Fungi	*Bacteroides fragilis*, _Z, NE, A_	97	Bacteria
Human Herpesvirus 1, _NZ, E_	171	Viruses	*Gibberella moniliformis*, _NZ, E_	97	Fungi
Respiratory Syncytial Virus, _NZ, NE, GBD_	164	Viruses	West Nile Virus, _Z, E, A, EC_	97	Viruses
Human Herpesvirus 5, _NZ, E_	159	Viruses	Human Herpesvirus 2, _NZ, E_	96	Viruses
*Haemophilus influenzae*, _NZ, E, GBD, EC_	148	Bacteria	Rabies Virus, _Z, E, A, GBD, EC_	96	Viruses
Lymphocytic Choriomeningitis Virus, _Z, NE, A_	148	Viruses	Hepatitis A Virus, _Z, E, GBD, EC_	95	Viruses
*Toxoplasma gondii*, _Z, E, A, EC_	148	Protozoa	Human Herpesvirus 6, _NZ, NE_	94	Viruses
*Klebsiella pneumoniae*, _Z, E, A_	146	Bacteria	*Fusarium oxysporum*, _NZ, E_	93	Fungi
*Vibrio cholerae*, _NZ, E, GBD, EC_	145	Bacteria	*Mycoplasma pneumoniae*, _NZ, NE_	93	Bacteria
*Borrelia burgdorferi*, _Z, E, A_	144	Bacteria	*Cryptosporidium parvum*, _Z, E, A, GBD, EC_	92	Protozoa
*Chlamydophila pneumoniae*, _Z, NE, A_	143	Bacteria	*Enterobacter cloacae*, _Z, NE, A_	90	Bacteria
*Shigella flexneri*, _Z, NE, GBD, EC_	142	Bacteria	*Aeromonas hydrophila*, _Z, E, A_	89	Bacteria
Human Herpesvirus 8, _NZ, E_	140	Viruses	*Acinetobacter baumannii*, _NZ, NE, A_	88	Bacteria
*Escherichia coli* o157∶h7, _Z, E, EC_	138	Bacteria	*Candida glabrata*, _Z, E_	87	Fungi
Human T-lymphotropic Virus 1, _NZ, E_	137	Viruses	*Moraxella catarrhalis*, _NZ, NE_	87	Bacteria
*Neisseria gonorrhoeae*, _NZ, E, GBD, EC_	136	Bacteria	*Salmonella enterica* subsp. *enterica* serovar enteritidis, _Z, E, A, GBD, EC_	87	Bacteria
Influenza A Virus, _Z, E, A, GBD, EC_	135	Viruses	*Treponema pallidum*, _NZ, E, GBD, EC_	87	Bacteria
*Legionella pneumophila*, _NZ, E, EC_	133	Bacteria	*Trichomonas vaginalis*, _NZ, E, GBD_	87	Protozoa
*Enterococcus faecalis*, _Z, E_	132	Bacteria	*Rhizopus oryzae*, _Z, NE_	86	Fungi
*Mycobacterium bovis*, _Z, E, A_	132	Bacteria	Hepatitis E Virus, _Z, E, GBD_	83	Viruses
*Campylobacter jejuni*, _Z, E, A, GBD, EC_	130	Bacteria	Human Parvovirus b19, _NZ, E_	81	Viruses
*Neisseria meningitidis*, _NZ, E, GBD, EC_	130	Bacteria	*Proteus mirabilis*, _Z, NE, A_	80	Bacteria
*Chlamydia trachomatis*, _Z, E, GBD, EC_	129	Bacteria	*Shigella dysenteriae*, _Z, E, GBD, EC_	80	Bacteria
*Clostridium difficile*, _Z, E, A_	127	Bacteria	*Stenotrophomonas maltophilia*, _NZ, NE_	80	Bacteria
*Cryptococcus neoformans*, _Z, E, A_	126	Fungi	*Bacillus licheniformis*, _Z, NE, A_	78	Bacteria
*Yersinia enterocolitica*, _Z, E, A_	126	Bacteria	*Mycoplasma genitalium*, _NZ, NE_	78	Bacteria
Mouse Mammary Tumor Virus, _Z, E, NS_	125	Viruses	*Trichinella spiralis*, _Z, E, A, EC_	78	Helminths
*Mycobacterium avium*, _Z, E_	125	Bacteria	*Bartonella henselae*, _Z, NE, A_	77	Bacteria
*Bacillus anthracis*, _Z, E, A, EC_	122	Bacteria	*Salmonella enterica* subsp. *enterica* serovar typhimurium, _Z, E, A, GBD, EC_	77	Bacteria
*Bordetella pertussis*, _NZ, E, GBD, EC_	122	Bacteria	*Brucella abortus*, _Z, E, A, EC_	76	Bacteria
Measles Virus, _Z, E, GBD, EC_	119	Viruses	*Candida tropicalis*, _Z, NE_	76	Fungi
Human Enterovirus C, _NZ, NE_	118	Viruses	*Pseudomonas stutzeri*, _NZ, NE_	76	Bacteria
*Enterococcus faecium*, _Z, E_	116	Bacteria	SARS coronavirus, _Z, E, NS, EC_	76	Viruses
*Staphylococcus epidermidis*, _Z, E, A_	114	Bacteria	*Enterobacter aerogenes*, _Z, NE, A_	75	Bacteria
Human Herpesvirus 3, _NZ, E, GBD_	113	Viruses	*Francisella tularensis*, _Z, E, A, EC_	74	Bacteria
*Porphyromonas gingivalis*, _Z, NE_	113	Bacteria	*Vibrio parahaemolyticus*, _Z, E_	74	Bacteria

Pathogens include those which are zoonotic (Z), non-zoonotic (NZ), emerging (E) and not emerging (NE) [15], [16], or given a new status (NS) in this work. Pathogens also included in the list of top 100 animal pathogens are noted (A). The major pathogens causing diseases included within the 2012 Global Burden of Disease (GBD) report are noted [8], [31], as are those reportable in the EC (EC) [9].

**Table 3:** Top 100 domestic animal pathogens in Europe, prioritised according to the H-index methodology [6] with the same emerging and zoonotic definitions as for Table 2.

Pathogen name	H-index score	Taxonomic division	Pathogen name	H-index score	Taxonomic division
*Escherichia coli*, _Z, E, H, DISC_	524	Bacteria	*Salmonella enterica* subsp. *enterica* serovar typhimurium, _Z, E, H, DISC_	77	Bacteria
*Staphylococcus aureus*, _Z, E, H_	271	Bacteria	*Brucella abortus*, _Z, E, H, OIE_	76	Bacteria
*Helicobacter pylori*, _Z, NE, H, DISC_	246	Bacteria	*Enterobacter aerogenes*, _Z, NE, H, DISC_	75	Bacteria
*Pseudomonas aeruginosa*, _Z, E, H_	243	Bacteria	*Francisella tularensis*, _Z, E, H, OIE_	74	Bacteria
*Listeria monocytogenes*, _Z, E, H_	207	Bacteria	*Haemonchus contortus*, _NZ, NE, NS_	74	Helminths
Murine Leukemia Virus, _Z, E, NS_	184	Viruses	*Neospora caninum*, _NZ, NE, NS_	72	Protozoa
*Candida albicans*, _Z, E, H, DISC_	181	Fungi	Cowpox Virus, _Z, E, NS_	71	Viruses
Lymphocytic Choriomeningitis Virus, _Z, NE, H_	148	Viruses	Bovine Herpesvirus 1, _NZ, NE, NS, OIE_	70	Viruses
*Toxoplasma gondii*, _Z, E, H, DISC_	148	Protozoa	*Citrobacter freundii*, _NZ, E, NS, DISC_	70	Bacteria
*Klebsiella pneumoniae*, _Z, E, H, DISC_	146	Bacteria	Feline Leukemia Virus, _NZ, NE, NS_	69	Viruses
*Borrelia burgdorferi*, _Z, E, H, DISC_	144	Bacteria	*Fasciola hepatica*, _Z, E, NS, DISC_	68	Helminths
*Chlamydophila pneumoniae*, _Z, NE, H, DISC_	143	Bacteria	Reticuloendotheliosis Virus, _NZ, NE, NS, DISC_	68	Viruses
Influenza A Virus, _Z, E, H, OIE_	135	Viruses	*Coxiella burnetii*, _Z, E, NS, OIE_	67	Bacteria
*Mycobacterium bovis*, _Z, E, H, OIE_	132	Bacteria	*Mannheimia haemolytica*, _Z, NE, NS, DISC_	67	Bacteria
*Campylobacter jejuni*, _Z, E, H, DISC_	130	Bacteria	Infectious Bronchitis Virus, _NZ, NE, NS, OIE_	66	Viruses
*Clostridium difficile*, _Z, E, H_	127	Bacteria	*Klebsiella oxytoca*, _NZ, NE, NS, DISC_	66	Bacteria
*Cryptococcus neoformans*, _Z, E, H, DISC_	126	Fungi	*Ascaris suum*, _Z, NE, NS_	65	Helminths
*Yersinia enterocolitica*, _Z, E, H_	126	Bacteria	Borna Disease Virus, _Z, E, NS, DISC_	65	Viruses
*Bacillus anthracis*, _Z, E, H, OIE_	122	Bacteria	Bovine Leukemia Virus, _NZ, NE, NS, DISC_	65	Viruses
*Staphylococcus epidermidis*, _Z, E, H, DISC_	114	Bacteria	*Campylobacter coli*, _Z, NE, NS, DISC_	65	Bacteria
*Bacillus cereus*, _Z, NE, H, DISC_	111	Bacteria	Canine Parvovirus, _NZ, NE, NS_	65	Viruses
*Clostridium botulinum*, _Z, E, H, DISC_	106	Bacteria	Parainfluenza Virus 5, _Z, NE, NS_	65	Viruses
Suid Herpesvirus 1, _NZ, NE, NS, OIE_	106	Viruses	*Pasteurella multocida*, _Z, NE, NS, OIE_	65	Bacteria
Encephalomyocarditis Virus, _Z, NE, H, DISC_	105	Viruses	Porcine circovirus, _NZ, E, NS, DISC_	65	Viruses
*Yersinia pestis*, _Z, E, H, DISC_	105	Bacteria	Porcine Reproductive and Respiratory Syndrome Virus, _NZ, E, NS, OIE_	65	Viruses
Bovine Spongiform Encephalopathy agent, _Z, E, NS, OIE_	101	Viruses	Bluetongue Virus, _NZ, E,NS, OIE_	64	Viruses
*Clostridium perfringens*, _Z, NE, H_	101	Bacteria	*Chlamydophila abortus*, _Z, E, NS, DISC_	63	Bacteria
*Yersinia pseudotuberculosis*, _Z, NE, H_	99	Bacteria	*Chlamydophila psittaci*, _Z, E, OIE, NS_	63	Bacteria
*Entamoeba histolytica*, _Z, NE, H, DISC_	98	Protozoa	*Enterococcus hirae*, _Z, NE, NS, DISC_	63	Bacteria
*Leishmania donovani*, _Z, E, H, OIE_	98	Protozoa	Gallid Herpesvirus 2, _NZ, NE, NS_	63	Viruses
*Bacteroides fragilis*, _Z, NE, H, DISC_	97	Bacteria	*Anaplasma phagocytophilum*, _Z, E, NS, OIE_	62	Bacteria
West Nile Virus, _Z, E, H, OIE_	97	Viruses	Equine Infectious Anemia Virus, _NZ, NE, NS, OIE_	62	Viruses
Rabies Virus, _Z, E, H, OIE_	96	Viruses	*Streptococcus agalactiae*, _Z, E, NS_	62	Bacteria
Bovine Papillomavirus, _NZ, E, NS, DISC_	95	Viruses	*Echinococcus granulosus*, _Z, E, NS, OIE_	61	Helminths
Newcastle Disease Virus, _Z, NE, NS, OIE_	93	Viruses	Equine Arteritis Virus, _NZ, E, NS, OIE_	61	Viruses
*Cryptosporidium parvum*, _Z, E, H_	92	Protozoa	Maedi Visna Virus, _NZ, NE, NS, OIE_	61	Viruses
*Enterobacter cloacae*, _Z, NE, H_	90	Bacteria	Canine Distemper Virus, _NZ, E, NS, DISC_	60	Viruses
*Aeromonas hydrophila*, _Z, E, H, DISC_	89	Bacteria	Chicken Anemia Virus, _NZ, E,NS, DISC_	60	Viruses
Foot and Mouth Disease Virus, _Z, NE, OIE_	89	Viruses	Equid Herpesvirus 1, _NZ, E, NS, OIE_	60	Viruses
*Acinetobacter baumannii*, _NZ, NE, H, DISC_	88	Bacteria	Infectious Bursal Disease Virus, _NZ, NE, NS, OIE_	60	Viruses
*Salmonella enterica* subsp. *enterica* serovar enteritidis, _Z, E, H, DISC_	87	Bacteria	Transmissible Gastroenteritis Virus, _NZ, NE, NS, OIE_	60	Viruses
Classical Swine Fever Virus, _NZ, NE, NS, OIE_	84	Viruses	*Actinobacillus pleuropneumoniae*, _NZ, E, NS, DISC_	59	Bacteria
Bovine Viral Diarrhea Virus 1, _NZ, E, NS, OIE_	80	Viruses	Feline Calicivirus, _Z, E, NS_	59	Viruses
Feline Immunodeficiency Virus, _NZ, E, NS_	80	Viruses	Myxoma Virus, _NZ, NE, NS, OIE_	59	Viruses
*Proteus mirabilis*, _Z, NE, H, DISC_	80	Bacteria	*Encephalitozoon cuniculi*, _Z, E, NS, DISC_	58	Fungi
*Mycobacterium avium* subsp. *paratuberculosis*, _NZ, E, NS, OIE_	79	Bacteria	Rotavirus A, _Z, E, NS, DISC_	58	Viruses
Scrapie agent, _NZ, NE, NS, OIE_	79	Viruses	*Campylobacter fetus*, _Z, E, NS_	57	Bacteria
*Bacillus licheniformis*, _Z, NE, H_	78	Bacteria	Fowlpox Virus, _NZ, NE, NS, DISC_	57	Viruses
*Trichinella spiralis*, _Z, E, H, OIE_	78	Helminths	*Leishmania infantum*, _Z, E, NS, OIE_	57	Protozoa
*Bartonella henselae*, _Z, NE, H, DISC_	77	Bacteria	*Trichostrongylus colubriformis*, _Z, NE, NS, DISC_	57	Helminths

Pathogens also included in the list of top 100 human pathogens are noted (H). The major pathogens causing diseases included within the OIE list of notifiable terrestrial and aquatic animal diseases (OIE) are noted [10], as are those included in the DISCONTOOLS project (DISC) [11].

### Data analyses

#### H-indices and previous prioritisations

Pearson's Chi-squared tests with Yates' continuity correction and Fisher's Exact Tests (FET) were used to test for differences in counts of pathogens included in previous work [9], [10], [11], according to outcomes including their taxonomic division, zoonotic and emerging status. Where appropriate, odds ratios (OR) and 95% confidence intervals (CI) are presented.

#### H-indices for One Health

Differences in H-indices for human-only, zoonotic, and animal-only pathogens were examined using a two-way Analysis of Variance (ANOVA) with log_10_-transformation of the response, including emerging status as an explanatory covariate. Post-hoc Tukey multiple comparisons of treatments were undertaken using the *HSD.test* [21].

#### H-indices for domestic animal pathogens

*-OIE list*. Homogeneity of variances of H-indices for animal-only (and not zoonotic) pathogens included or not within the OIE list of notifiable animal diseases was examined using the Fligner-Killeen (median) test. One-way ANOVA thereafter established differences in the (log_10_-transformed) H-indices of pathogens included or not in the OIE list. –*DISCONTOOLS*. H-indices and DISCONTOOLS scores were compared using Spearman's Rank correlations. If more than one pathogen had been included within disease information for the DISCONTOOLS rankings (for *Campylobacter*, Leishmaniasis, and Salmonellosis), the higher H-index score was used for analyses.

All analyses were undertaken using the statistical software package R [22], with statistical significance determined by a *P*-value of less than 0.05.

## Results

### Priority lists of human and domestic animal pathogens present in Europe

Two lists each including the top 100 human (Table 2) and domestic animal pathogens (Table 3) which cause significant clinical disease and which therefore need consideration from a health and well-being perspective were short-listed using the H-index prioritisation method (for alternative names and synonyms see [19]). When combined, 114 (72.6%) pathogens appeared solely in the human or animal list, and 43 (27.4%) were in both lists. Of the top 100 human pathogens, 66 were classed as zoonotic and 67 were emerging, compared to 67 and 57 for domestic animal pathogens, respectively.

### H-indices and previous prioritisations

Of the top 100 human pathogens identified, 42 were either included in the GBD [8], or are reportable to the EC [9], or both. Reasons for failure to include pathogens may be that pathogenic agents cause rarely diagnosed disease (e.g. Human T-lymphotropic Virus 1, Lymphocytic Choriomeningitis Virus, and *Moraxella catarrhalis*), or because disease agents are diverse, e.g. pneumonia or other lung infections (*Aspergillus niger*, *Chlamydophila pneumonia*, *Cryptococcus neoformans*, *Klebsiella pneumonia*, and *Mycoplasma pneumoniae*) and gastro-intestinal (GI) symptoms or GI-tract infections (*Aeromonas hydrophila*, *Bacillus cereus*, *Bacteroides fragilis*, *Clostridium species*, *Vibrio parahaemolyticus*, and *Yersinia enterocolitica*). The impact of chronic disease or diseases causing low morbidity may be difficult to quantify or seen as less important (*Bartonella henselae*, *Borrelia burgdorferi*, Human Enterovirus C, Human Herpesvirus group, Human Papillomavirus, Human Parvovirus b19, *Mycobacterium bovis*, *Mycobacterium avium*, and *Mycoplasma genitalium*). In addition, some pathogens may generally be commensals or natural biota (*Aggregatibacter actinomycetemcomitan*s, *Candida* species, *Enterobacter*, *Enterococcus*, *Staphylococcus* species, *Candida tropicalis*, *Helicobacter pylori*, and *Porphyromonas gingivalis*) or species existing in the environment (*Acinetobacter baumannii*, *Burkholderia cepacia*, *Candida glabrata*, *Entamoeba histolytica*, *Fusarium oxysporum*, *Gibberella moniliformis*, *Proteus mirabilis*, *Pseudomonas* species, *Rhizopus oryzae*, *Serratia marcescens*, *Staphylococcus aureus*, and *Stenotrophomonas maltophilia*) causing opportunistic infections in immune-compromised individuals (including those young, old or pregnant); their impact upon the general population may not be quantified.

Of the top 100 domestic animal pathogens described, 76 were either notifiable according to the OIE [10], or included in DISCONTOOLS [11], or both. Reasons for failure to include may be similar to for human pathogens (only pathogens not previously mentioned are cited: multiple disease symptoms or lack of diagnosis – *Ascaris suum*, Feline Immunodeficiency Virus, Feline Leukemia Virus, Gallid Herpesvirus 2, *Haemonchus contortus*, and *Yersinia pseudotuberculosis*; causes of specific disease being diverse, for respiratory infection - Feline Calicivirus, and GI symptoms - *Campylobacter fetus*, *Cryptosporidium parvum*, *and Listeria monocytogenes*,; and existing in the environment and opportunistic - *Pseudomonas aeruginosa*). In addition, some omitted pathogens may be production issues with impact difficult to quantify (*Streptococcus agalactiae* causing Mastitis in cattle and *Neospora caninum* causing abortion in cattle and dogs) and some may be issues of pets (Canine Parvovirus, *Neospora caninum*, and Parainfluenza Virus 5).

For human pathogens in our list (Table 2), fungi and helminths are particularly under-represented in GBD assessments (percentage included in GBD: bacteria, 25%; fungi, 0%; helminths, 0%; protozoa, 60%; viruses, 42.3%; FET, *P* = 0·046). There was no difference between taxonomic divisions in the percentage reportable to the EC (FET, *P* = 0.109). Human pathogens classed as emerging (compared to not emerging) were statistically more likely to have GBD estimates (FET, *P*<0.001, OR = 10.27, CI = 2.28–95.77) and be EC reportable (FET, *P*<0.001, OR = 16.00, CI = 3.49–144.92), but not more likely to have GBD estimates or be EC reportable if they were zoonotic compared to non-zoonotic (Pearson's χ^2^, *P* = 0.219 and FET, *P* = 0.745, respectively).

For domestic animal pathogens in our list (Table 3), fungi were particularly under-represented in the OIE list (percentage included in OIE: bacteria, 20%; fungi, 0%; helminths, 33.3%, protozoa, 33.3%, viruses, 52.5%; FET, *P* = 0.014). By contrast, viruses are particularly under-represented in DISCONTOOLS (percentage included in DISCONTOOLS: bacteria, 55.6%; fungi, 100%; helminths, 33.3%; protozoa, 33.3%; viruses, 25%; FET, *P* = 0.008). Animal pathogens classed as zoonotic (compared to non-zoonotic) were (of borderline statistical significance) less likely to be included in the OIE list (Pearson's χ^2^, *P* = 0.055, effect size(ø) = 0.22, OR = 0.39) but there was no difference in the percentage included in DISCONTOOLS, (Pearson's χ^2^, *P* = 0.309) nor any effect of being emerging (versus not emerging) on inclusion in either list (Pearson's χ^2^, *P* = 0.633 and *P*<0.99, respectively).

### H-indices for One Health

There was a statistically significant difference between the H-indices of zoonotic, human-only or animal-only pathogens (Two-way ANOVA, *F*_2,152_ = 24·40, *P*<0.001); H-indices were significantly higher for human-only (untransformed mean = 132.39±6.14 and lower for animal-only pathogens (68.11±6.06) compared to zoonotic (100.83±9.93). H-indices were higher (with borderline statistical significance) for emerging (106.61±10.19) compared to not emerging (86.91±8.14) pathogens (Two-way ANOVA, *F_1_*_,152_ = 3.78, *P* = 0.054). The interaction between zoonotic and emerging factors was not significant (*P* = 0.25).

### H-indices for domestic animal pathogens

#### -OIE list

There was no difference in the H-indices of animal-only pathogens included or not within the OIE list (ANOVA *F*(1, 31) = 0·005, *MSE*<0.001, *P* = 0.943; variances not significantly different by Fligner-Killeen (median) test.

#### -DISCONTOOLS

There were significant correlations between H-indices and DISCONTOOLS estimates of public (human) health (zoonotic and animal pathogens) and impact on wider society (animal-only pathogens), and a further relationship of borderline significance between H-indices and the DISCONTOOLS overall result; no other correlations were significant (Table 4).

**Table 4:** Results of Spearman's Rank correlations between H-indices and the DISCONTOOLS prioritisation of major animal diseases [11].

	Zoonotic and animal-only pathogens		Animal-only pathogens	
Subsection of prioritisation	S rho	*P* value	S rho	*P* value
Disease knowledge	0·004	0·987	−0·180	0·699
Impact on animal health and welfare	−0·048	0·830	0·309	0·500
Impact on public (human) health	0·449	**0·032**	0·586	0·166
Impact on wider society	0·081	0·713	−0·775	**0·041**
Impact on trade	0·200	0·360	−0·216	0·641
Control tools	0·170	0·438	0·093	0·842
Overall results	0·379	0·074*	>0·001	<0·999

Notations include Spearman's Rank correlation value (S rho) and *P* values for the correlation. *P* values of significance are shown in bold, and a relationship of borderline significance is marked *.

## Discussion

The European Commission has outlined measures to strengthen coordinated approaches to health security at EU level, including monitoring, early warning and combating specific threats of a cross-border nature. These measures could be for climate change resilience [23] or for biosecurity, particularly for infectious diseases including communicable diseases, antimicrobial resistant and healthcare-associated infections related to communicable diseases, and biotoxins or other biological agents [24]. In this study, we implement a number of previously defined actions [25], including presenting a quantitative evaluation for the impact of infectious pathogens affecting human health and well-being (via effects upon domestic animals) [24]. The work is unique, starting with all known infectious pathogens, and then objectively and systematically deciding which occur in relevant hosts in Europe using a transparent process. The study establishes priority lists of human and domestic animal pathogens (including zoonoses) present in Europe, using the H-index as a proxy measure for impact.

Previous work suggests that higher H-indices indicate higher impact for a pathogen relative to lower H-indices [6], (McIntyre, *unpublished material*). The H-index method has both strengths and weaknesses. The strengths include that it is much more evidence-based and objective than semi-quantitative and qualitative approaches, and the results provide an easily understood quantitative estimate of impact. H-indices estimates can be simply and rapidly calculated, and they can therefore be repeatedly obtained to reflect changes in status, with the potential for automation of this process. The results are available for all pathogens at a global scale, and the scores reflect the wider scientific interest that would be expected to follow from a pathogen being either zoonotic or emerging [6]. Most importantly, within a study of 27 human diseases, H-indices were correlated with DALY estimates [6], (McIntyre, *unpublished material*). DALYs are an accepted measure of true disease burden in humans which accounts for the years of healthy life lost as a result of poor health or disability as well as the potential years of life lost due to premature death [26]. In further work, H-indices were also correlated with the number of human deaths (McIntyre, *unpublished material*).

The weaknesses of the H-index method include that calculations need some manual oversight, as false positives can occur for instance when pathogens are used as model organisms; biases in results may happen because of trends in interest in specific pathogens, diseases or research fields or in certain regions; and estimates are subject to biases in funding (McIntyre, *unpublished material*) and research publication. H-indices are likely to underestimate the contribution of scientific literature published in non-English languages, although after translation some publications are included in WoS and consequently in our calculations of H-indices. The literature searching method also doesn't account for the quality of publications in which pathogen names appear and the typical number of citations within different fields, and all bibliographic software packages incorporate newly published literature from different literature sources into their databases at different rates. Finally, H-indices are only a proxy for impact, with the results susceptible to a lag in time-to-publication, and newly emerging pathogens likely to be under-represented.

As the strengths and weaknesses of using the H-index method are different to those of other prioritisation methods, it is probably best used in combination with other approaches, for example, to shortlist a set of pathogens for more detailed risk assessment relying on expert opinion or qualitative data. It may also be used to prioritise diseases for which formal risk assessments are not possible because of data gaps.

Our priority lists of pathogens enabled investigation of why infectious pathogens are omitted from disease surveillance and impact quantification work [8], [9], [10], [11]. We considered several reasons for exclusion, including lack of diagnosis or misdiagnosis [27], because the impact of particularly chronic infections is difficult to quantify or they are seen as less important, and because some pathogens are commensals or natural biota causing opportunistic infections in immune-compromised individuals; their impact upon the general population may therefore not be quantified. Recently discovered pathogens may be under-represented as a sufficient body of evidence for their impact may not have been accrued within the literature, for example Norovirus infection does not appear within the top 100 human pathogens. In addition, domestic pet pathogens, particularly if non-zoonotic or easily controlled, may not be included. Further analysis suggests that pathogens fail to be included dependent upon their taxonomic division (perhaps due to diagnostic issues) and for human pathogens, if they are not emerging, perhaps as a result of the ‘Matthew effect’ (‘the rich get richer and the poor get poorer’); equally, they may simply have less long-term impact. The effect of emerging status is unsurprising, given this focus in surveillance work and despite a lag in time-to-publication leading to their likely under-representation in H-index calculations [6]; for some animal pathogens, this is the first time that emerging status has been examined.

Methods to assess disease impacts use metrics capturing either human or animal host effects; they neither measure the magnitude in all hosts nor take account of scientific knowledge and tools for control. It is hard to prioritise human and animal diseases, because of the different metrics used (health or societal impacts versus welfare or economic impacts). Significant differences between H-indices mean values for human-only, zoonotic, and animal-only pathogens provide evidence that this single measure may have some use as a One Health metric accounting for such factors. For example values for zoonotic pathogens were higher than for animal-only, suggesting that they account for human as well as animal-impact. Higher values for human-only compared to other pathogen groups suggests that zoonoses may be under-represented due to underestimation of their global burden [28], [29], or research impact [6], or because of biases in research impact and funding for chronic human pathogens [29]. In addition, lower animal-only H-indices may be due to funding biases.

Finally, there was limited evidence that the H-index method is a reasonable proxy for the impact of animal pathogens; animal pathogen H-indices were significantly positively correlated with subsections of DISCONTOOLS [11], including impact on public (human) health and overall results (borderline significance). If animal-only (not zoonotic) diseases were included, there was a significant positive relationship with impact on wider society. As the more animal-focussed subsections (disease knowledge, impact on animal health and welfare, impact on trade, and available control tools) were not correlated with H-indices, and H-indices were not affected by inclusion in the OIE list [10], this suggests a human-centric bias in H-indices; for example, a pathogen causing little impact in animals may nevertheless have a high H-index if zoonotic.

The priority lists presented in this work should be used by agencies and research organisations in combination with other risk assessment methods to identify gaps in working for priority setting. It has been suggested that zoonoses must be dealt with at the interface of human and animal health using all available information [30]; this work, combining the EID2 and H-index technique, demonstrates such ‘big-data’ approaches.
